# Plyometric Exercise Transiently Enhances Twitch Torque but Fails to Enhance the Rate of Force Development Evaluated Using the Isometric Midthigh Pull

**DOI:** 10.5114/jhk/186979

**Published:** 2024-07-17

**Authors:** Kaito Nakata, Takaaki Mishima

**Affiliations:** 1 Graduate School of Sport and Exercise Sciences, Osaka University of Health and Sport Sciences, Sennan, Osaka, Japan.; 2 Osaka University of Health and Sport Sciences, School of Health and Sport Sciences, Sennan, Osaka, Japan.

**Keywords:** post-activation potentiation, post-activation performance enhancement, countermovement jump

## Abstract

The effects of jump exercises as a conditioning activity (CA) on the rate of force development (RFD) measured during the isometric midthigh pull (IMTP) have not been investigated. Therefore, we aimed to investigate the effect of the CA comprising three sets of five countermovement jumps on the RFD measured during the IMTP, and furthermore to investigate whether post-activity potentiation (PAP) would be involved in this mechanism. Thirteen healthy male university students (age: 19.7 ± 0.6 years; training experience: 3.0 ± 1.4 years) participated in five sessions. Initially, the twitch torque was elicited by electrical stimulation after the CA to investigate whether PAP was elicited. Participants then completed the following four sessions: experimental condition sessions, in which the IMTP was performed 1 or 4 min after the CA, and control condition sessions, in which the IMTP was performed 7 or 10 min after the pre-measurement because the experimental condition included a 2-min rest interval before the CA and approximately 4 min were required to perform the CA. One-way analysis of variance (ANOVA) with Bonferroni post-hoc adjustments revealed the presence of PAP 1 and 2 min after the CA. Two-way ANOVA revealed significant interaction effects for the 0–200 ms and 0–250 ms RFD at the 1st min, and the 0–150 ms and 0–250 ms RFD at the 4th min after the CA. However, the Bonferroni post-hoc test failed to detect any significant increase in the RFD index under the experimental conditions. The CA with jump exercises induced PAP up to 2 min after the CA implementation, but failed to significantly increase the RFD measured by the IMTP.

## Introduction

Post-activation performance enhancement (PAPE) refers to the phenomenon of transient improvement in voluntary exercise performance, such as jumping or sprinting, after performing a voluntary conditioning activity (CA) at a high intensity (Cuenca-Fernández et al., 2017). The main mechanism underlying PAPE is suggested to be the involvement of post-activation potentiation (PAP), referring to twitch torque evoked by a single electrical stimulus that increases transiently after a maximal or a submaximal CA (Hamada et al., 2000); phosphorylation of myosin regulatory light chains may be involved in this mechanism (Sweeney et al., 1993). Furthermore, mechanisms such as potentiation of selected neuromuscular responses, heightened muscle temperature, and alterations in water content within the muscle may be involved in PAPE (Blazevich and Babault, 2019). Numerous athletes and strength coaches aspire to efficiently optimize performance within a limited number of training sessions. A previous study reported that exercises performed under conditions that transiently benefit from potentiating effects may produce long-term supplemental performance gains over time (Talpey et al., 2016). Moreover, in elite sports, where marginal performance differences affect competition outcomes, incorporating effective CA immediately preceding competitive performance may be desirable.

Several studies have reported that plyometric exercises may augment voluntary performance (Ciocca et al., 2021; Kümmel et al., 2018; Seitz and Haff, 2016; Zimmermann et al., 2021). Employing a CA in plyometric exercises does not require equipment and can be integrated effortlessly in pre-competition warm-ups. Several studies have investigated changes in athletic performance using jump exercises as a CA (Ciocca et al., 2021; Kümmel et al., 2018; Zimmermann et al., 2021; Chamera et al., 2023); however, whether jump exercise as a CA affects the rate of force development (RFD) remains unclear (Zimmermann et al., 2022). The RFD constitutes a comprehensive metric closely linked to performance across sports, and an improved RFD following jump exercises as a CA could support the use of the CA as a warm-up. However, Zimmermann et al. (2022) reported that after performing three sets of five countermovement jumps (CMJs) as a CA, the RFD measured during isometric knee extension did not increase significantly in any of the rest intervals from 2 to 10 min post-CA. Nonetheless, single-joint RFD measurements may not fully reflect the outcomes of multi-joint movements observed in actual athletic performance. An early study reported no significant correlation between the RFD measured in knee extension exercises and CMJ performance (Baker et al., 1994). Therefore, while single-joint tasks provide a controlled environment for assessing the underlying physiological determinants of the RFD, multi-joint tasks that more closely resemble sporting situations are recommended to be more useful for practical results (Maffiuletti et al., 2016).

The isometric midthigh pull (IMTP) is a multi-joint isometric assessment executed in a second pull-starting posture, such as the clean or the snatch. The RFD assessed using the IMTP serves as a significant marker for diverse athletic performances (Wang et al., 2016). Hence, any augmentation in the RFD detected during the IMTP following jump exercises as a CA could potentially show the impact of the CA on athletic performance. Noteworthy, the RFD measured in isometric contractions is improved by neurological factors including more activated motor units, lower recruitment thresholds, and greater motor unit discharge rates (Maffiuletti et al., 2016). Importantly, plyometric exercises, which elicit a stretch reflex, acutely increase excitation potential transmittance via Ia afferents, increasing motor neuron pool output and resulting in higher-order motor unit activation during subsequent activity (Tillin and Bishop., 2009). It is, thus, possible that plyometric exercises may improve the RFD measured during the IMTP via neurological factors. However, to the best of our knowledge, no studies have investigated the effects of jump exercises as a CA on the RFD measured during the IMTP. Although PAP associated with the CA is considered a main mechanism of PAPE, a recent report suggested that PAP might not necessarily be the only mechanism underlying PAPE (Blazevich and Babault, 2019). Thus, whether PAP is involved in the mechanism of PAPE after jump exercise as a CA remains unclear.

Therefore, we aimed to determine whether jump exercises as a CA would enhance the RFD measured during the subsequent IMTP. In addition, we elicited twitch torque using electrical stimulation after the CA to investigate whether PAP was involved. We hypothesized that jump exercises used as a CA would transiently affect the RFD measured during the IMTP, with an involvement of PAP. This study should provide more practical information on transiently improving sports performance and training quality.

## Methods

### Study Design

**Figure 1:**
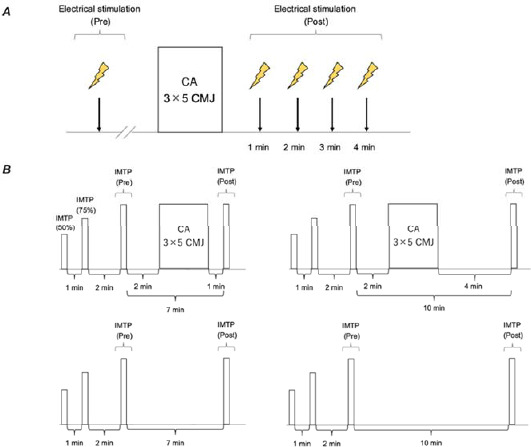
Study overview. Familiarization session procedure (A). Experiment session procedure (B). CA, conditioning activity; IMTP, isometric midthigh pull; CMJ, countermovement jump

This study involved five sessions per participant. The initial session observed PAP using electric stimulation of the quadriceps femoris through the implementation of the CA and familiarized participants with the IMTP (Figure 1A). Sessions 2–5 investigated the influence of the CA on the RFD. The experimental condition comprised the CA performed 2 min after baseline measurements. One or four min later, the IMTP was reperformed as a post-measurement. As the total time to complete the CA was about 4 min, the post-measurement under the control condition was performed after 7 or 10 min of passive rest to match the time when the CA was performed under the experimental condition (Figure 1B). All participants underwent four randomly scheduled sessions on separate days. In addition, a minimum rest period of 24 h was included between all sessions. The experiment was conducted in an indoor laboratory setting from 10:00 AM to 3:00 PM.

### Participants

Thirteen healthy male university students participated in this study (mean ± standard deviation; age 19.7 ± 0.6 years; body height 174.1 ± 4.6 cm; body mass 70.8 ± 3.4 kg; training experience 3.0 ± 1.4 years). G*Power software (v3.1) was used to determine sample sizes using the following variables: medium effect size (f = 0.25), the alpha level of 0.05, and the power level of 0.8. A minimum sample size of 12 individuals was obtained. We recruited 13 participants to account for sample loss. All participants had a minimum of one year of resistance training experience and had not sustained lower limb orthopedic injuries for at least six months prior to the study. During the experimental period, participants were instructed to maintain their usual daily activities and to refrain from altering their levels of physical activity, dietary intake, or fluid consumption. Furthermore, participants were advised to abstain from the intake of depressants (e.g., alcohol) and ergogenic substances (e.g., caffeine) for 24 h before the experiment. Prior to the experiment, participants received verbal and written explanations of the purpose, possible risks, and procedures. All experiments were conducted after obtaining informed consent. This study was approved by the Ethics Committee of the Osaka University of Health and Sport Sciences (approval number: 21-31; approval date: 17 February 2022).

### Design and Procedures

#### Jump Exercises

We adopted the CA protocol from Zimmermann et al. (2022), incorporating a regimen of three sets of five CMJs. Participants were instructed to keep their hands on their waist, bend their knees to nearly 90° during the descending phase, and strive to achieve maximal height during the jump. An inter-set rest of 1 min was provided.

#### Electrical Stimulation Procedure

The initial session aimed to observe temporal changes in PAP following the CA. After arriving at the laboratory, participants were required to limit physical activity for 10 min and remain at rest to eliminate the effects of PAP induced during travel to the laboratory. PAP declines quasi-exponentially and becomes negligible after approximately 5 min (Hamada et al., 2000). Two electrode pads consisting of 10 × 20-cm aluminum foil with an adhesive conductor (SR-4080; Minato Medical Science, Osaka, Japan) used to evoke quadriceps muscle twitches were attached to the upper and the lower anterior thigh of the dominant leg. Participants were then placed on a dynamometer (Biodex System 4; Sakai Medical Instrument, Tokyo, Japan) with fixed knee (90°) and hip (80°) joints and were tightly secured to the seat using two crossover seatbelts and a waist harness. The lever arm of the dynamometer was attached 2–3 cm above the lateral malleolus using a strap. The rotational axis of the knee was aligned with the motor axis. Electrode pads were connected to a high-voltage constant-current stimulator (DS7AH; Digitimer Ltd., Hertfordshire, UK) via an output cable (D185-HB4; Digitimer Ltd., Hertfordshire, UK). The stimulus intensity was set at 120% of the maximum intensity (Hamada et al., 2003). After assessing the isometric peak twitch torque at baseline, participants were released from the dynamometer and executed the CA. Next, participants were firmly resecured to the dynamometer. Twitch torque was then evoked at temporal intervals of 1, 2, 3, and 4 min after the CA. Twitch torque at the 1^st^ and the 4^th^ min after the CA was used to investigate whether PAP was related to the PAPE after the same rest interval under the experimental conditions. The peak twitch contraction of the dynamometer was passed through an A/D converter (Power Lab/8SP; ADInstruments, Bella Vista, NSW, Australia) and stored (LabChart 6 Japanese; ADInstruments). Data were filtered (low-pass Butterworth filter, cut-off frequency of 12 Hz), with the cut-off frequency determined from preliminary experiments. Specifically, it was determined by confirming that three standard deviations of the noise were not greater than the threshold of 0.5 N for the contraction onset of isometric knee extension (Maffiuletti et al., 2016). The sampling frequency was set to 100 Hz. The extent of PAP measured during the initial session was expressed as %PAP, calculated as follows:


$$\%PAP=\left(\frac{Post-Pre}{Pre}\right)\times 100$$

where *Pre* and *Post* are the maximal twitch torque induced before and after the CA, respectively.

### Procedures for Determining the Effect of the CA on the RFD

**Figure 2:**
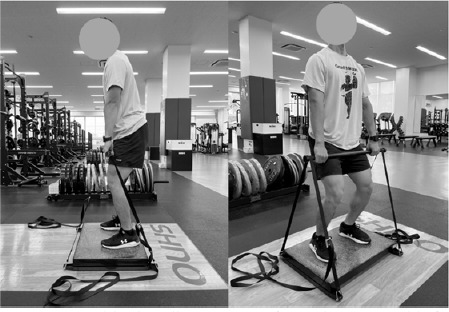
Isometric midthigh pull position performed on a portable force plate.

The details of sessions 2–5 are shown in Figure 1B. Initially, participants engaged in a warm-up encompassing 5-min treadmill running at 5 km/h, followed by 10-min of rest. Then, they performed the IMTP for 3 s at 50% and 75% of their subjective maximum effort as a baseline warm-up, with a 1-min rest interval between warm-up sets. After the warm-up, participants performed one IMTP at 100% of their subjective maximal effort as a baseline measurement, followed by a 2-min rest interval and the CA with jump exercises under the experimental condition. Under the experimental condition, the IMTP was performed after 1 or 4 min of rest after the CA, while under the control condition, the IMTP was performed after 7 or 10 min of passive rest without physical activity from the baseline IMTP. The IMTP was performed on a portable force plate (C-Force; Innervations, Columbus, Perth, Australia) (Figure 2). The IMTP was performed using the hip and knee joints at 145° and 140°, respectively. The IMTP at this joint angle is considered similar to the second pull of a snatch or the clean and may exert the greatest force throughout the lifting phase (Brady et al., 2020). Participants were instructed to apply a small amount of tension and then pull on the bar as hard and as fast as possible for 5 s with strong verbal encouragement for maximal effort. In all tests, participants wore wrist wraps. The peak RFD, 0–150 ms RFD, 0–200 ms RFD, and 0–250 ms RFD were measured. The reliability of the RFD was assessed in our preliminary study using the intraclass correlation coefficient (ICC) (two-way mixed-effects, average measures, absolute agreement); with an ICC of the peak RFD, 0–150 ms RFD, 0–200 ms RFD, and 0–250 ms RFD of 0.947 (95% confidence interval [CI]: 0.736–0.989), 0.831 (95% CI: 0.158–0.966), 0.907 (95% CI: 0.535–0.981), and 0.965 (95% CI: 0.824–0.993), respectively.

### Statistical Analysis

Descriptive data are presented as mean ± standard deviation. Data normality was examined using the Shapiro-Wilk test. The %PAP after the CA was tested using a one-way repeated-measures analysis of variance (ANOVA). Variance homogeneity was examined using the Mauchly sphericity test for repeated measures; if homogeneity was violated, Greenhouse-Geisser correction was applied for further analyses. When a significant main effect was found, the Bonferroni post-hoc test was used to examine time-course changes in %PAP. A two-way ANOVA (time [Pre, Post] × condition [control condition, experimental condition]) was used to assess the effects of the CA on the RFD measured by the IMTP, with the Bonferroni post-hoc test used for significant interactions. Effect sizes were estimated using partial eta-squared (*η*^2^) values (small: 0.01–0.059, moderate: 0.06–0.137, and large: >0.138). For pairwise comparisons, effect size was determined using Cohen’s *d* (small, >0.2; moderate, >0.5; large, >0.8) (Cohen, 1988). All statistical analyses were performed using SPSS software (v27.0; IBM Corp., Armonk, NY, USA). Significance was set at *p* < 0.05, and the threshold for trends of statistical significance was set at *p* < 0.10.

## Results

### Twitch Torque

**Figure 3:**
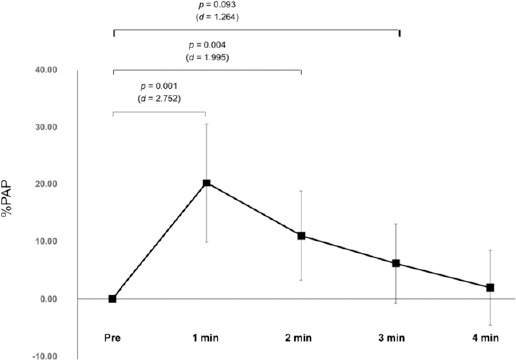
Time-course changes in %PAP after the CA. Statistical significance was set at p < 0.05. The trend for statistical significance was set at p < 0.10

Twitch torque evoked by electrical stimulation following CMJs is shown in Figure 3. One-way ANOVA revealed a significant main effect (*p* = 0.001, *η*^2^ = 0.806). The peak twitch torque values at the 1^st^ min (+20.2 ± 10.4%, *p* = 0.001, *d* = 2.752, 95% CI: 9.9–30.5) and the 2^nd^ min (+110.0 ± 7.8%, *p* = 0.004, *d* = 1.995, 95% CI: 3.3–18.7) were significantly higher than those at baseline. In addition, a trend towards potentiation was observed at the 3^rd^ min (+6.1 ± 6.9%, *p* = 0.093, *d* = 1.264, 95% CI: −0.7–12.9) after the CA, compared with the baseline value.

### RFD Measured by the IMTP

**Figure 4:**
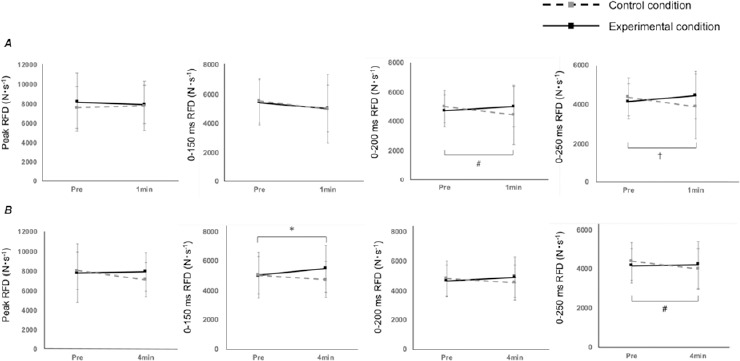
Time course of the peak RFD for each of the experimental and control conditions. Values are presented as means ± standard deviation. The solid and dotted lines represent the experimental and control conditions, respectively. Statistical tendency from Pre values: * p < 0.10 under the experimental condition, ^†^ p < 0.10 under the control condition. Significant difference from Pre values: ^#^ p < 0.05 under the control condition

Changes in the RFD observed during the IMTP following the CA are shown in Figure 4. Two-way ANOVA revealed significant time × condition interactions at the 1^st^ min after the CA for the 0–200 ms RFD (*p* = 0.032, *η*^2^ = 0.178) and for the 0–250 ms RFD (*p* = 0.034, *η*^2^ = 0.175). Further analyses showed significantly lower values for the 0–200 ms RFD under the control condition than the Pre values (−575.6 ± 268.6 N·s^−1^, *p* = 0.042, *d* = 0.355, 95% CI: −1130.0 to −21.2). A statistical trend towards lower values was observed for the 0–250 ms RFD under the control condition when compared with the Pre values (−484.5 ± 251.5 N·s^−1^, *p* = 0.067, *d* = 0.356, 95% CI: −1002.5–35.6). Two-way ANOVA revealed that time × condition interactions were significant at the 4^th^ min after the CA for the 0–150 ms RFD (*p* = 0.04, *η*^2^ = 0.165) and for the 0–250 ms RFD (*p* = 0.033, *η*^2^ = 0.176). Further analyses showed a statistical trend towards increased values for the 0–150 ms RFD under the experimental condition when compared with Pre values (+455.7 ± 244.8 N·s^−1^, *p* = 0.075, *d* = 0.247, 95%CI: −49.5 to 960.9). Conversely, the 0–250 ms RFD under the control condition showed significantly lower values compared with the Pre values (−396.5 ± 358.2 N·s^−1^, *p* = 0.010, *d* = 0.396, 95% CI: −6890.0 to −104.1).

## Discussion

This study investigated whether the CMJ would influence the RFD measured using the IMTP and whether PAP would be involved. We hypothesized that jump exercises such as CA would transiently affect the RFD measured during the IMTP and that PAP would be involved. PAP was observed between the 1^st^ and the 2^nd^ min after the CA completion, while none of the RFD indices obtained using the IMTP assessed 1 and 4 min after the CMJ completion exhibited statistically significant increases; thus, the initial hypothesis was rejected.

Zimmermann et al. (2021) showed improvements in 30-m sprint times at the 2^nd^ and the 4^th^ min after three sets of five CMJs. The discrepancies between the present and previous findings may be caused by the differences in the exercises performed after the CA. Specifically, although jumping exercise as the CA is effective for sports movements involving stretch-shortening cycles, its effect may be limited to movements involving isometric contractions, as performed herein. Indeed, Kümmel et al. (2018) found that the execution of 10 repetitive reactive hops subsequently enhanced drop jump performance, suggesting involvement of changes in the behavior of the muscle-tendon units during stretch-shortening cycles. Prior plyometric exercise diminished the elongation of muscle fibers during the eccentric phase, suggesting that it may concurrently augment elastic energy in passive elastic structures during this phase. Thus, it is possible that jump exercises as a CA improve sports performance involving the stretch-shortening cycle, with only a limited effect on isometric contractions.

Notably, the 0–150 ms RFD measured 4 min after CMJs as a CA showed a trend toward being higher than the corresponding Pre values (Figure 4B). In contrast, PAP measured 4 min after the CA had already disappeared (Figure 3), suggesting that PAP was not involved in this enhancement. Similarly, Nakata and Mishima (2022) confirmed the presence of PAPE 4–7 min after the CA using knee extension exercises; however, PAP had already disappeared by that time. Blazevich and Babault (2019) indicated that factors other than PAP, such as an increase in muscle temperature and changes in central drive capacity, may contribute to acute voluntary performance enhancement. Importantly, an early RFD (e.g., 0–150 ms RFD) measured in isometric contractions was improved by neurological factors including more activated motor units, lower recruitment thresholds, and greater motor unit discharge rates (Maffiuletti et al., 2016). Interestingly, plyometric exercises can elicit stretch reflex responses, which increase the excitation potential transmittance via Ia afferents. This increases output from motor neuron pools and results in higher-order motor unit activation during subsequent activity (Tillin and Bishop., 2009). Therefore, the observed trend of the improved RFD in the 0–150 ms window could be attributed to neurological factors.

Figure 3 shows that three sets of five CMJs increased twitch torque evoked by electrical stimulation up to 2 min after the CA, which is consistent with the findings of Zimmermann et al. (2022). In contrast, none of the RFD indices showed significant changes at the 1^st^ min after the CA in the presence of PAP (Figure 4A). Several studies have indicated that PAP is not always associated with PAPE (Blazevich and Babault, 2019; Nakata and Mishima, 2022; Zimmermann et al., 2020, 2022). Importantly, sensitivity to the potentiation effect of the CA differs between twitch torque and maximal voluntary torque (Sweeney et al., 1993). Ca^2+^ concentration is much higher in maximal voluntary concentric contractions (multiple impulses) than in twitch contractions (single impulse). In addition, the extent of the increase in torque by the CA decreases when Ca^2+^ concentration is higher because several cross-bridge attachments already exist (Persechini et al., 1985). Zimmermann et al. (2020) noted that PAP must be extremely high to contribute to an increase in voluntary performance. Consequently, it is possible that the degree of PAP observed in the present study (1 min, 20.2%) was insufficient to enhance voluntary exercise performance.

Notably, under the control condition, RFDs at the 7^th^ min (0–200 ms) after the baseline measurement and at the 10^th^ min (0–250 ms) were significantly lower than those at baseline (Figure 4A and B); this may be related to decreased muscle temperature. Fluctuations in muscle temperature have a significant impact on explosive performance, with a 1°C change resulting in a 2–5% change in explosive performance (McGowan et al., 2015). Importantly, muscle temperature may be higher than baseline values for up to 15–20 min after physical exercise (McGowan et al., 2015). Therefore, the baseline values in the present study may reflect the effects of increased muscle temperature due to the treadmill-based warm-up performed 10 min prior. However, under the control condition, an additional rest interval (7 or 10 min) was provided after the baseline IMTP, which would have additionally decreased muscle temperature and possibly, the RFD. Notably, Mohr et al. (2004) reported a 2.0°C decrease in muscle temperature when a 15-min half-time period was provided after the first half of a soccer game, which may have been associated with decreased sprint performance measured just before the second half. However, they also found that rewarm-up during half-time prevented decreases in muscle temperature and did not decrease sprint performance. In the present study, it is possible that the experimental condition prevented decreases in muscle temperature because of the CA implementation.

This study has some limitations. First, the study employed only two rest intervals from the CA (1 and 4 min). Several studies have demonstrated that a rest interval of 4 min is sufficient to induce PAPE (Kümmel et al., 2018; Zimmermann et al., 2021), while others have reported that a longer rest interval is necessary (Seitz and Haff, 2016). In the future, investigations can focus on whether PAPE occurs after a longer rest time after the implementation of the CA using the CMJ. We also point out that most of the participants in the present study were athletes who began training after entering college. Several studies have reported that the effects of PAPE are greater in athletes with greater muscle strength than in those with lower levels of strength (Nakata and Mishima, 2022; Seitz and Haff, 2016). Therefore, investigating the effectiveness of the CA using the CMJ in athletes with a higher level of muscular strength is desirable.

## Conclusions

In the present study, a CA consisting of jumping exercises elicited PAP up to 2 min after the CA, but failed to increase the RFD measured by subsequent isometric contraction at either time point, even when performed with multi-joint exercises. The present results should not be interpreted as indicating that jump exercise as a CA is not effective at inducing PAPE. It is possible that the potentiating effects of jump exercises as a CA may have a specific positive effect on exercises involving stretch-shortening cycles, which are frequently observed in sports. Therefore, future studies should examine whether the effects on voluntary performance after a CA performed with plyometric exercises differ depending on the contraction mode of the testing task (i.e., static vs. dynamic), and investigate the potentiation mechanisms by simultaneously examining PAP.
